# An Update of the Sanguinarine and Benzophenanthridine Alkaloids’ Biosynthesis and Their Applications

**DOI:** 10.3390/molecules27041378

**Published:** 2022-02-18

**Authors:** José Ignacio Laines-Hidalgo, José Armando Muñoz-Sánchez, Lloyd Loza-Müller, Felipe Vázquez-Flota

**Affiliations:** Unidad de Bioquímica y Biología Molecular de Plantas, Centro de Investigación Científica de Yucatán, Calle 43 No. 130, Chuburná, Merida 97205, Mexico; jose.laines@cicy.mx (J.I.L.-H.); arms@cicy.mx (J.A.M.-S.); lloyd.loza@cicy.mx (L.L.-M.)

**Keywords:** alkaloids, benzylisoquinoline, benzophenanthridines, natural products, specialized metabolism

## Abstract

Benzophenanthridines belong to the benzylisoquinolic alkaloids, representing one of the main groups of this class. These alkaloids include over 120 different compounds, mostly in plants from the Fumariaceae, Papaveraceae, and Rutaceae families, which confer chemical protection against pathogens and herbivores. Industrial uses of BZD include the production of environmentally friendly agrochemicals and livestock food supplements. However, although mainly considered toxic compounds, plants bearing them have been used in traditional medicine and their medical applications as antimicrobials, antiprotozoals, and cytotoxic agents have been envisioned. The biosynthetic pathways for some BZD have been established in different species, allowing for the isolation of the genes and enzymes involved. This knowledge has resulted in a better understanding of the process controlling their synthesis and an opening of the gates towards their exploitation by applying modern biotechnological approaches, such as synthetic biology. This review presents the new advances on BDZ biosynthesis and physiological roles. Industrial applications, mainly with pharmacological approaches, are also revised.

## 1. Introduction

**Figure 1:**
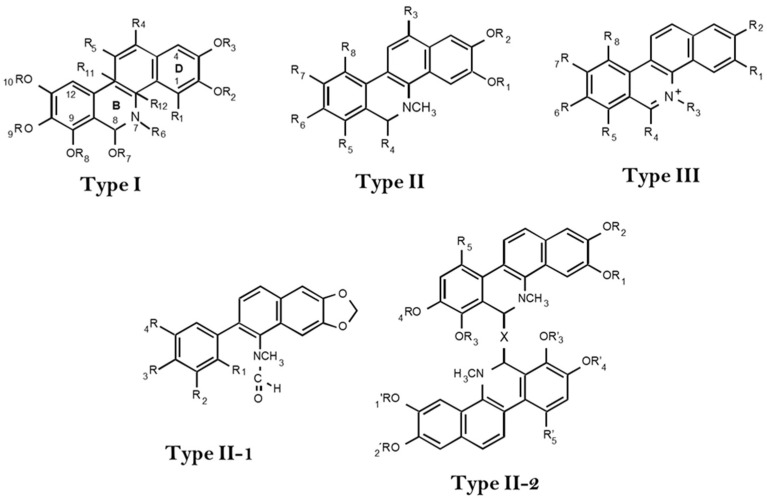
Chemical structure of the different benzophenathridines (BZD).

Benzophenanthridines (BZD) represent one of the 11 classes of benzylisoquinoline alkaloids (BIA). BZD include over 120 alkaloids mainly spread in plants from the Fumariaceae, Papaveraceae, and Rutaceae families, within the Ranunculales and Sapindales orders [1]. Structurally, BZD are tetracyclic compounds, which include a non-aromatic heterocyclic system (B ring). Depending on the ring organization, BZD could be classified in three different groups: Type I (hexahydrobenzo[c]phenanthridines) includes two aromatic (A and D) and aliphatic (B and C) rings, in which the N atom in B is usually methylated. In BZD type II (dihydrobenzo[c]phenanthridines), the N atom is also methylated; however, ring B could be absent when the C_7_-C_8_ bond is open (type II-1) or modified with a complex substituent at C_8_ (type II-2). Regardless of ring B being absent, rings A, C, and D are aromatic systems. Finally, BDZ type III results from the N protonation of BDZ type I or II, leading to the corresponding ammonium quaternary salts (Figure 1) [2,3]. Depending on the chemical modifications within these basic structures, up to seven groups can be distinguished: hexahydrobenzophenanthridines (type I), seco-benzophenanthridines (type II-1), dimeric dihydrobenzophenanthridines (type II-2), *N*-demethylbenzophenanthridines, dihydrobenzophenanthridines, benzophenanthridones, and quaternary benzophenanthridine alkaloids (type III; Figure 1) [2,3].

**Figure 2:**
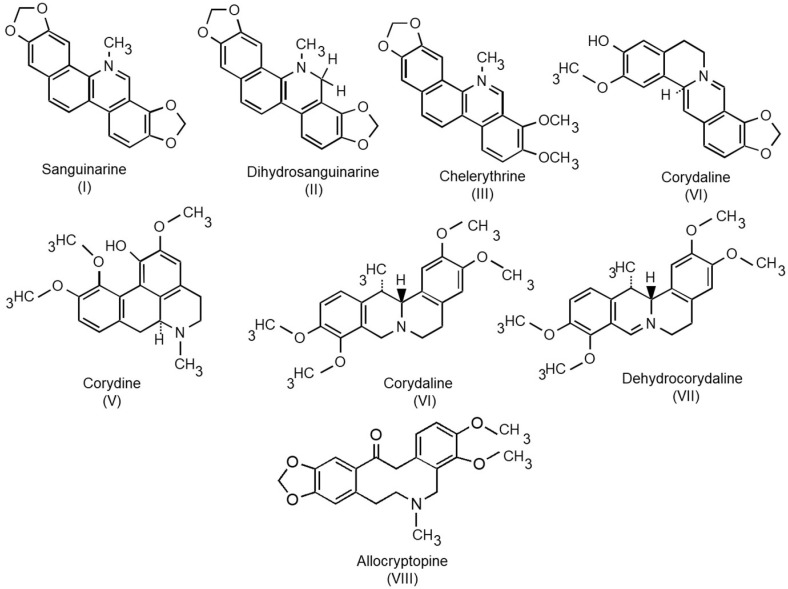
Chemical structure of the BZD analyzed in this review.

Plants bearing BDZ have had important roles in human traditional medicine in both the old and new world cultures for centuries. For example, the great celandine (*Chelidonium majus* L.; Papaveraceae) was used by ancient Greeks to treat eye cataracts, whereas the fern leafed corydalis (*Corydalis cheilanthifolia* Helms; Fumarioideae/Papaveraceae) is mentioned in antique Chinese treatises for irregular menses. In the Americas, prior to Europeans’ arrival, the prickly poppy (*Argemone mexicana* L., Papaveraceae) was recommended to remove genital warts and for other skin infections [4]. Some of the alkaloids found in these plants are shown in Figure 2 and include sanguinarine (I) and its reduced dihydro form, dihydrosanguinarine (II), chelerythrine (III), cheilanthifoline (IV), corydine (V), corydaline (VI), and dehydrocorydaline (VII), among others. Table 1 lists some plant species that accumulate in BZD alkaloids as well as their different uses for medicinal purposes. There is an increasing interest in sanguinarine and other BZD alkaloids due to their diverse pharmacological effects and possible functions in plant–environment interactions. Here, a review of the new advances on the BDZ biosynthetic process and physiological roles is presented. Industrial applications, mainly with pharmacological approaches, are also revised.

## 2. The Biosynthesis of Sanguinarine and Related BZD: Physiological Roles and Applications

The following sections present a view on the most relevant advancements on the topic.

### 2.1. Synthesis and Regulation of Sanguinarine and Related Alkaloids

#### 2.1.1. The Biosynthetic Pathway

**Figure 3:**
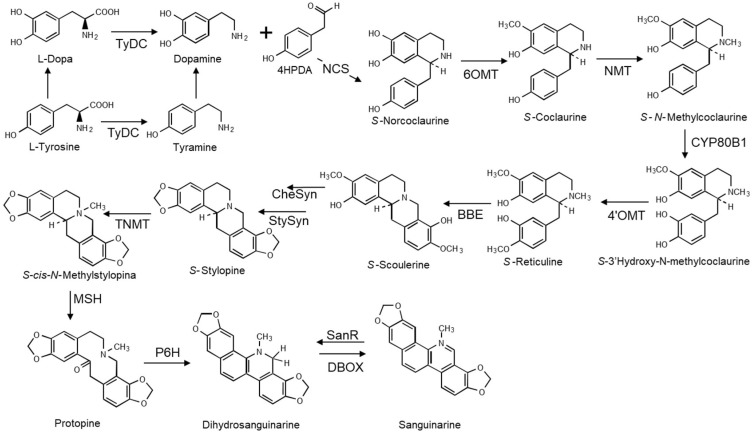
Biosynthetic pathways leading to sanguinarine. TyDC, l-tyrosine decarboxylase; NCS, s-norcoclaurine synthase; 6OMT, s-norcoclaurine-6-*O*-methyltransferase; BBE, reticuline oxidase: berberine bridge enzyme; CheSyn, cheilanthifoline synthase; StySyn, stylopine synthase; TNMT, s-tetrahydroprotoberberine N-methyltransferase; MSH, methyltetrahydroprotoberberine 14-monooxygenase; P6H, protopine 6-hydroxylase; DBOX, dihydrobenzophenanthridine oxidase; and SanR, sanguinarine reductase.

The synthesis of BZD shares initial reactions with other BIA (Figure 3), starting with two units of l-tyrosine. One of them is decarboxylated, forming tyramine, which is then hydroxylated into dopamine. Alternatively, 3,4-dihydroxyphenylalanine (l-dopa) could be directly decarboxylated. Regardless of how dopamine is formed, it is later condensed with 4-hydroxyphenylacetaldehyde (4HPDA), which is the result of tyramine deamination, producing s-norcoclaurine, the central trihydroxylated intermediary from which all the wide BIA diversity will rise (Figure 3). Enzymes involved in these reactions are shown in Table 2. Among those, norcoclaurine synthase (NCS) has been studied in different species, displaying a positive substrate-binding typical of limiting enzymes [12]. Norcoclaurine is transformed in s-reticuline after an ordered series of reactions involving O-6 methylation, N methylation, C3′ hydroxylation, and O-4′ methylation (Figure 3 and Table 2). Although the purified enzymes involved in these reactions could accept structurally related substrates in in vitro assays, all of them display a marked preference for BIA intermediaries [13]. Besides, alternative products could be formed in some species, which results in nearly 300 different alkaloids (Figure 3). This catalytic flexibility, associated to a recent evolutive divergence, is one of the reasons for BIA diversity and the restricted occurrence of different BIA types to a few related species [14].

Reticuline represents another important branching point, leading, on one hand, to the formation of morphinan and protomorphinan alkaloids, and to the groups of protoberberines, secoberberines, protopines, and BZD [14]. The first reaction in this set is the formation of the methylene bridge at C8, producing s-scoulerine, catalyzed in a stereo-specific manner by the berberine bridge enzyme (BBE; Figure 2), which is rapidly induced in response to pathogen and chemical elicitors, and thus related to plant defense in different species [15]. Oxidation of methoxy groups on rings A and D form the corresponding methylendioxy bridges rendering s-chelantifoline (III) and *s*-stylopine, respectively (Figure 3) [16,17]. N-methylation of stylopine to *s*-N-methylstylopine and its further oxidation produces protopine, which is then 6-hydroxylated, driving it to the spontaneous opening of the C ring as well as to the rearrangement and dehydration yielding dihydrosanguinarine (II), the first BDZ alkaloid, which is then oxidized to sanguinarine (I) [13] (Figure 3 and Table 2). This biosynthetic route, proposed earlier by the trace of radiolabeled precursors [18], has been confirmed by biochemical and genetic studies, including high throughput approaches in different species such as *Argemone mexicana* [17], *Corydalis yanhuso* [6], *Eschscholzia californica* [19], *Macleaya cordata* [8], and *Papaver somniferum* [13], among others. However, species-associated particularities have been noticed in some cases. For example, in *A. mexicana*, CYP719A13 encodes a cytochrome P450 with activity of both stylopine and canadine synthase since it can accept cheilanthifoline (IV) as well as tetrahydrocolumbamine as substrates, transforming them into stylopine and canadine, respectively, and hence participating in sanguinarine (I) and berberine synthesis [17]. Moreover, enzymes involved in the formation of other BZD, such as chelerythrine (III), have also been identified by combining transcriptomic and metabolomic approaches [8]. Figure 3 depicts the reactions involved in the synthesis of selected BZD alkaloids, showing the acronyms for the enzymes involved in each reaction. Table 2 lists details of the identified enzymes.

#### 2.1.2. Tissue Distribution and Regulation

Commonly, sanguinarine (I) and other BZD alkaloids are synthetized in underground tissues in species of *Argemone*, *Papaver*, and *Sanguinaria,* where enzymes are distributed between the smooth endoplasmic reticulum (SER), both inside in the lumen and membrane-bound, and the cytosol [16,20]. After their synthesis, alkaloids are accumulated in vacuoles with the participation of a vesicle-mediated transport system [21]. In fact, this vesicle-mediated transport system allows for the mobilization of intermediaries, as they are being modified by the membrane-bound enzymes, in the track from SER to vacuole. Moreover, conditions promoting sanguinarine (I) synthesis also concomitantly induce major SER ultrastructural modifications [22].

However, both synthesis and accumulation could also take place in aerial tissues in *Bocconia*, *Chelidonium*, and *Macleaya* [8,23]. Mobilization of sanguinarine (I) through ABCB-type transporters has been determined in *A. mexicana* and *E. californica.* EcABCB1 was preferentially expressed in the roots of *E*. *californica*, which is the major site of accumulation for this alkaloid [24]. Interestingly, AmABCB1 from *A*. *mexicana* was reported mainly to be expressed in seeds with an important expression also observed in roots and in very low levels in leaves [25]. The specific role that sanguinarine could have in seeds of *A*. *mexicana* is unknown but it is suggested that it might act as a defense against herbivores due to its high toxicity.

Genes involved in the biosynthesis of sanguinarine (I) and other BIA have been reported under transcriptional regulation of different elements, such as basic Helix-Loop-Helix (bHLH) and WRKY proteins. In *E*. *californica*, a number of sanguinarine biosynthetic genes were under the control of two bHLH proteins, named EcbHLH1-1 and -2, whereas tetrahydroprotoberberine cis-N-methyltransferase (TNMT) was regulated by EcbHLH1-1 and EcbHLH1-2-controlled 4′OMT. Interestingly, 6′OMT and stylopine synthase CYP719A3 (Figure 2) were regulated by both proteins [26]. The heterologous expression of a *Coptis japonica* WRKY protein (CjWRKY) in *E. californica*-cultured cells increased the production of sanguinarine and 14 other components, and this was related to an increased expression of biosynthetic genes [27].

### 2.2. Physiological Roles of Benzophenanthridines

Specialized metabolites play critical roles in plant fitness to their surroundings. While some of them can act to attract pollen and seed dispersers, others exert toxic effects to vertebrate and invertebrate animals, fungi, and bacteria. As other plant alkaloids, BZD display toxic effects against herbivores [28] and different soil bacteria and fungi [29]. Sanguinarine has been the most studied among BZD alkaloids, displaying both herbivore deterrent activity as well as antimicrobial effects. Structural features conferring these physiological effects are related to BZD planar configuration, which allows them to intercalate in nucleic acids, affecting both DNA and RNA synthesis. Besides, as heteroaromatic iminium cations, BZD could bind to negatively charged membrane surfaces and proteins, and react with SH-compounds, interfering, in such a way, with the function of several cytosolic and membranal proteins, such as collagenase, tubulin assembly, and Na^+^/K^+^ ATPases, among others [30].

Sanguinarine (I), as other alkaloids, modifies its chemical behavior depending on the external pH. It acts as a polar, hydrophilic iminium cation or as a lipophilic, uncharged alkaloamine at pH values lower or higher than six, respectively [2]. In this way, both forms could coexist at a physiological pH. Interestingly, although bioactivity is associated with the iminium form, the low polar alkanolamine exhibits membrane permeability, which allows the alkaloid free movement across organelle membranes. Once inside the compartment’s lumen, interaction with an acidic milieu, such as the presence of a nucleic acid, produces an equilibrium shift towards the iminium active form [2,30]. Alternating these chemical forms ensures movement of the inactive, low toxic forms, which would turn into active ones once the target is reached, without participation of protein receptors.

#### 2.2.1. Herbivore Deterrence of Benzophenanthridines

Sanguinarine (I) interferes with the nerve impulse transmission in arthropods and vertebrates due to its notable inhibitory effects on choline acetyltransferase activity (IC_50_ 284 nM). It also hinders nicotinergic, muscarinergic, and serotonin-2 receptors, impairing, in this way, nerve transmission [10,31,32]. In addition to these effects, sanguinarine also reduces the feeding activity of *Lymantria dispar* (Lepidoptera) larvae on *C. majus* plants (LD_50_ of 4.963 μg/larva). Overall, these responses, which eventually lead to larvae mortality, were associated to the inhibition of digestive enzymes, such as α-amylase, lipase, and serine protease at the transcriptional level [33].

Interestingly, herbivory induces an increase in the contents of sanguinarine in plants, such as *E. californica* [34], *Chelidonium majus* [33], *M. cordata* [29], and *Sanguinaria canadensis* [35]. Other BZD, such as allocryptopine (VIII), cheilanthifoline (IV), and chelerythrine (III) with similar insecticidal effects, although to a lesser extent, were also augmented in some of these species, suggesting a common effect of BZD on predators’ nerve impulses and herbivore deterrence.

#### 2.2.2. Antimicrobial Activity of Benzophenanthridines

Most studies on BZD antimicrobial activity are related to their potential as pharmaceuticals with relatively few published works on their direct role in plant chemical defense. Different fungi-caused diseases, such as powdery mildew, blight, and root rot, have been observed in plants producing BZD alkaloids, including *Eschscholzia* [36], *Macleaya* [37,38], and *Papaver* [35,39], among others. *Fusarium oxysporum* and different species of *Erysiphe, Dendryphion*, and *Pleospora* have been identified as the etiological agents. Interestingly, even when sanguinarine (I), chelerythrine, and other BZD show antifungal activities [40,41], the direct effects of these fungi infections on alkaloid biosynthesis have not been analyzed in planta, despite abundant studies in in vitro cultures [42]. For instance, cell cultures of *A. mexicana* respond to the addition of *F. oxysporum* homogenates by increasing sanguinarine formation after activation of the octadecanoic signaling pathway [43,44]. Similar responses have also been noted in *Eschscholzia* [21] and *Papaver* [1], among others. Moreover, complex signaling pathways leading to the activation of alkaloid synthesis, which involve heterotrimeric G proteins, phospholipase A_2_, and cytoplasmic pH changes, have been established in cell cultures, suggesting an important role of these alkaloids in chemical defense [45,46].

Sanguinarine (I), along with chelerythrine (III) and corydaline (VI) to a lesser extent, displayed fungicidal activity against eight phytopathogenic fungi, including *Botrytis* *cinerea*, *Fusarium graminearum*, *F. oxysporum*, and *Magnaporthe oryzae*. These BZD-induced morphological abnormalities in mycelia resulted from deformed hyphae that eventually collapsed due to the disruption of membranes’ integrity. Moreover, an upsurge of reactive oxygen species (ROS) was observed in fungi exposed to BZD, mainly to sanguinarine, and it was associated to alterations in the redox potential of mitochondrial membranes and modifications in the nuclear morphology [47]. These hyphal deformations in *M. oryzae* resulted in a lower appressorium tissue penetration in barley and disturbed the proper germ tubes’ formation from spores. Such effects are derived from the interference of sanguinarine (I) with the fungus cAMP-mediated signaling pathways [48] and could explain the antifungal effects.

Sanguinarine (I) and chelerythrine (III) also showed antimicrobial effects against soil-borne pathogenic bacteria, such as *Agrobacterium tumefaciens*, *Pseudomonas lachrymans*, and *Xanthomonas vesicatoria* [49]. These effects are mainly due to alkaloid interference in the assembly of the FtsZ protein, a tubulin homologous, into filaments that conform the cytokinesis contracting belt, thus hampering bacterial fission. A direct binding of alkaloids’ dimethoxy and isoquinoline groups to the protein domains involved in polymerization has been observed [50].

Interestingly, even when different pathogenic viruses have been isolated from *Papaver* species, which accumulates sanguinarine [51,52], no studies on the effect of such infections on alkaloid synthesis have been reported.

### 2.3. Medical and Industrial Applications of Benzophenanthridines

Perhaps the main industrial use of BDZ in commercial products is the manufacturing of livestock feed additives from *M. cordata* (Sangrovit^®^; Phytobiotics Futterzusatzstoffe GmbH; Eltville am Rhein, Germany). The product is sold as pellets or pearls made from dried plant material that contain sanguinarine, protopine, and chelerythrine in no less than 60% on a dry weight basis and it is claimed to improve food digestibility in broilers, swine, cattle, sheep, and farmed fisheries. These effects have been related mainly to alkaloids, helping to control intestinal infections [53]. Interestingly, sanguinarine (I) inhibits the formation of biofilms by different enterobacteria, such as carbapenem-resistant *Serratia marcescens* and *Providencia rettgeri* [54,55]. Additionally, sanguinarine could avoid inflammation and retard amino acid transit by inhibiting amino acid decarboxylases’ action [56]. Until recently, antiplaque mouth washes and toothpaste containing extracts from *S. canadensis* were commercialized (Viadent^®^; Colgate-Palmolive Inc, New York City, NY, USA), but they are no longer available [11]. Extracts of *A. mexicana* are mixed with other plants in the elaboration of ecofriendly insecticides (BioDie; Promotora Técnica Industrial SA, Jiutepec México) [4]. Noteworthy is that, although mainly considered toxic compounds [57], BZD alkaloids also display similar pharmaceutical effects, although with different efficacy, due to the same structural features exposed above. BZD exhibit hypotensive, antimicrobial, antioxidant, and anti-inflammatory properties [56]. Moreover, cytotoxic and cytostatic effects on different human cancer cells have also been reported [30]. No cell receptors for these alkaloids have been described; hence, their physiological activity depends on their diffusion into cells and chemical reactivity with membranes, nucleic acids, and proteins, and their consequent interference in different biochemical processes. For example, the disruption of membrane electrochemical gradients, caused by inhibition of Na^+^/K^+^ ATPases, also hampers different signal transduction pathways, including those mediated by mitogen-activating protein kinases (MAPK), reactive oxygen species (ROS), and intracellular calcium, which, in turn, could be involved in cell death and apoptosis pathways [58]. It has been observed that chelerythrine, chelidonine (stylophorin), cordatine, and nitidine induce ROS formation in tumoral cell lines from diverse human tissues, including bladder, breast, glioma, lung, kidney, pancreas, prostate, skin, stomach, and uvea, among others [30,57,58,59]. ROS accumulation induced by BZD activates different components of the apoptotic transduction pathways, including both caspase-dependent and independent routes [58]. Such an increase in ROS cell levels is therapeutically valued since recent anticancer approaches are aimed at agents that trigger a controlled upsurge, leading to the activation of the antioxidant cell response, which, in turn, would attenuate tumor development [30,59,60]. Besides these pleiotropic effects and nucleic acid intercalation, direct interaction with enzymes, such as DNA pol, topoisomerases, etc., and membrane lipid peroxidation also account for bioactivity. Increased ROS levels, induced by BZD, also have additional beneficial medical implications for type 2 diabetes (T2D) treatment. Extracts from *Fumaria parviflora* have shown hypoglycemic effects in rats, which were associated to sanguinarine contents, and bioinformatic prospections pulled out sanguinarine (I) in the top 20 out 6100 candidates with alleged antidiabetic properties [61]. Recently, sanguinarine has been shown to modulate ROS accumulation in kidney tissues of streptozotocine-induced diabetic rats. This not only reduced the expression of tumoral markers but also resulted in protection against diabetic nephropathy [62]. These activities rely on their structural features. Structural requirements for activity include planar configuration, N methyl substitutions, hydroxylation patterns, and the fusion of the B/C ring [2,3,57].

Sanguinarine (I), chelerythrine (III), and their direct dihydro derivatives are the most studied alkaloids among BZD. However, other minor compounds also display similar physiological activities, although with wide variations in their effective doses [2,3]. Table 3 shows some of the main BZD alkaloids with their ascribed pharmacological activity and the biochemical mechanisms involved.

## 3. Concluding Remarks

In plants, BDZ alkaloids are part of an elaborated chemical defensive system sensitive to potentially harmful environmental stimuli, such as microbial infections and insect foraging. Interaction with these biological agents sets up a chain of biochemical events leading to the transcriptional activation of genes involved in their synthesis and mobilization [21,44,45,46,47]. The defensive role of secondary metabolites, such as alkaloids, in plants has turned into a valuable biotechnological tool, which is frequently used in in vitro cell cultures to increase their accumulation. Cell cultures submitted to conditions mimicking microbial infection or environmental hazards respond by increasing transcriptional activity, leading to the synthesis of these defensive compounds (the process is known as elicitation) [77]. Cell cultures from different species from the Papaveraceae family [42], including *A. mexicana* [78], *E. californica* [21], *M. cordata* [8], and *P. somniferum* [79], show this response when challenged by exposition to different stimuli. Besides its potential for commercial exploitation, elicitation has also allowed for the discovery of new enzymes [42,80] and has led to the design of ingenious approaches for chemical semisynthetic processes. For example, recently, an interest on BZD dihydro forms, mainly dihydrosanguinarine (II), has surged due to its minor cytotoxicity [81]. In plants, sanguinarine reductase (SanR; Figure 3) leads to the NADH-dependent reduction of sanguinarine and other BZD to the lesser toxic dihydro derivatives. This is part of a mechanism to avoid cell damages caused by an increase in the alkaloid accumulation in response to pathogen infection [21]. This response has instigated the development of a biomimetic approach for the reduction of the N_7_=C_8_ double-bond located in the B ring by incubating sanguinarine (I) with NADH under 455 nm blue radiation. This reaction produced a dihydrosanguinarine (II) dimer but also can be applied for the semi-synthesis of different natural substituted dihydroBZD [81]. In fact, an efficient total synthesis of selected BZD, such as chelerythrine (III) and sanguinarine (I), has been recently reported using affordable materials, such as 7-azabenzonorbornadiene and based on enzyme mechanisms [82]. However, although well-established at the cellular level, details on the operation of these mechanisms in integral tissues or the whole plant are yet to be discovered. Moreover, the complete biosynthetic pathway required for sanguinarine (I) and other BZD alkaloids has been isolated from different species [6,8,16,19], and some regulatory genes, and their corresponding cis-elements are also available [26,27]. This has allowed for dissecting which components of the pathway are responsive to specific biochemical mediators [45] and the mechanisms involved in this response. On the other hand, although still under observation due to undesirable side effects, the medical applications of sanguinarine (I) and related alkaloids is an area of intensive research (Table 3). Interestingly, BZD have been considered both cancerogenic and anticancer agents [83]. Epidemiological evidence links the use of mouthwashes added with sanguinarine to maxillary vestibule oral leukoplakia as well as its involuntary consumption in contaminated mustard oil to gall bladder [11,83]. However, different genotoxicity assays and animal tests for cancer genesis often deliver non-conclusive results. Therefore, neither sanguinarine (I) nor other BZD are currently listed as proved cancer-producing agents [83]. The BZD planar structure is similar to other polyaromatic hydrocarbon carcinogens that allow for intercalation in the DNA [2,3]. Besides, BZD induce cell oxidative burst with ROS formation. Paradoxically, although ROS accumulation triggered by BZD could cause DNA damage, it also could be the basis of their therapeutical application against cancer. This may be explained by the fact that a controlled increase in ROS could induce the activation of internal cell mechanisms directed to the mitigation of oxidative harm [58,59]. Moreover, sanguinarine and other BZD inhibited the P-glycoprotein/ABCB1 and related ABCB5 in drug resistance in multidrug-resistant tumor lines, increasing their sensitivity to cytotoxic drugs, which might allow for better treatments [84]. In this way, knowledge generated on the synthesis and regulation of these alkaloids could now be directed towards the generation of tools for their commercial exploitation by modern biotechnological methods, including cell culture technology and synthetic biology approaches. Scaling up for massive culture of elicited cell suspension has been reported [79] and the introduction of the complete sanguinarine (I) pathway to yeast cells has been recently achieved [85]. Moreover, the availability of the complete set of genes involved in sanguinarine (I) synthesis, as well as some of the regulatory genes and those involved in its mobilization, would allow for improving not only the enzymatic catalysis by gene edition but also the internal cell traffic of intermediaries, resulting in more efficient processes for the formation of these valuable alkaloids.

## Figures and Tables

**Table 1:** A few plants bearing BZD alkaloids and their traditional medicinal uses.

Plant Species	Applications	References
*Argemone mexicana* L. (Papaveraceae)	Antiprotozoals: to dissolve eye cataracts, to remove warts, and to treat skin infections	[4]
*Chelidonium majus* L. (Papaveraceae)	Skin, liver, and eye diseases; antiparasitic	[5]
*Corydalis yanhusuo* Chou (Papaveraceae)	Analgesic for chest pain, post-partum blood stasis, and spleen and stomach stasis	[6,7]
*Macleaya cordata* Willd (Papaveraceae)	Anti-inflammatory and antimicrobial activities	[8]
*Eschscholzia californica* Cham (Papaveraceae)	Sedative, anxiolytic, analgesic, soporific, spasmolytic, diuretic, and diaphoretic	[9,10]
*Sanguinaria canadensis* L. (Papaveraceae)	To treat cold and congestion, sore throats, emetic, abdominal cramps, lumps, wound infections, and rheumatism	[11]

**Table 2:** Enzymes involved in sanguinarine biosynthesis, describing reaction catalyzed and subcellular localization. Data were collected from UniProt Beta (https://beta.uniprot.org/ (accessed on 12 December 2021)).

Enzyme and EC Number	Function	Subcellular Localization	Organisms amd Acc. Number
l-Tyrosine decarboxylase (TyDC) EC 4.1.1.25	Decarboxylates of l-tyrosine to produce tyramine	Cytosol	*P. somniferum* (P54768) *Thalictrum flavum* (Q9AXN7) *A. mexicana* (D2SMM8)
s-Norcoclaurine synthase (NCS) EC 4.2.1.78	Condenses dopamine and 4-HPDA, producing s-norcoclaurine	Endoplasmic reticulum lumen and vacuole	*P. somniferum* (Q4QTJ2) *T. flavum* (Q67A25) *A. mexicana* (EU881891)
*s*-Norcoclaurine-6-*O*-methyltransferase (6OMT) EC 2.1.1.128	Transfers a methyl group from SAM to s-norcoclaurine, forming coclaurine, and to r,s-norcoclaurine, formimg r-norprotosinomenine, s-norprotosinomenine, and (r,s)-isoorientaline	Membrane integral protein	*P. somniferum* (Q6WUC1) *Coptis japonica* (Q9LEL6)
Reticuline oxidase: berberine bridge enzyme (BBE) EC:1.21.3.3	Converts s-reticuline in s-scoulerine by forming of a carbon–carbon bond (C8) between the *N*-methyl group and the phenolic ring	Cytoplasmic vesicles	*P. somniferum* (P93479) *E. californica* (P30986) *A. mexicana* (D2SMM9)
Cheilanthifoline synthase (CheSyn) EC:1.14.19.65	Converts s-scoulerine into r,s-cheilanthifoline by forming a methylenedioxy brigde	Endoplasmic reticulum membrane	*E. californica* (B5UAQ8) *A. mexicana* (CYP719A14)
Stylopine synthase (StySyn) EC:1.14.19.73	Forms a methylenedioxy bridge on ring A (2,3 position), transforming s-cheilanthifoline to s-stylopine, s-scoulerine to s-nandinine, and s-tetrahydrocolumbamine to s-canadine	Smooth endoplasmic reticulum membrane	*E. californica* (Q50LH4) *A. mexicana* (B1NF19)
s-Tetrahydroprotoberberine N-methyltransferase TNMT EC:2.1.1.122	Converts stylopine, canadine, and tetrahydropalmatine to their corresponding N-methylated products	Cytosol	*P. somniferum* (Q108P1) *E. californica* C3SBS8
Methyltetrahydroprotoberberine 14-monooxygenase (MSH) EC:1.14.14.97	Transforms, by oxidation, N-methylstylopine and N-methylcanadine into protopine and allocryptopine, respectively	Membranal protein	*P. somniferum* (L7X3S1)
Protopine 6-hydroxylase (P6H) EC:1.14.14.98	Converts protopine and allocryptopine to dihydrosanguinarine and dihydrochelerythrine by hydroxylation at position 6	Endoplasmic reticulum membrane	*E. californica* (F2Z9C1) *P. somniferum* (L7X0L7)
Dihydrobenzophenanthridine oxidase (DBOX) EC 1.5.3.12	Catalyzes a two-electron oxidation of dihydrosanguinarine, forming sanguinarine	Endoplasmic reticulum	*P. somniferum* (AAC61839)
Sanguinarine reductase (SanR) EC:1.3.1.107	Catalyzes reduction of benzophenanthridines, preferentially sanguinarine, to the dihydroalkaloids; involved in detoxifying the phytoalexins produced by plant itself	Vacoule	*E. californica* (D5JWB3)

**Table 3:** Pharmacological effects of sanguinarine and related alkaloids.

Alkaloid	Effects	Mechanism	References
Sanguinarine (I)	Antimicrobial	Halts formation of contracting belt by binding to the FtsZ protein	[50,63]
		Interferes with carbohydrate metabolisms by inhibiting glucose transport and the 2-ketogluconate pathway	
		Increases sensitivity to β-lactam antibiotics	
	Antiretroviral	Inhibits transcriptase reverse	[64]
	Anticancer	Cytotoxic Intercalates DNA and RNA, affecting topoisomerase action and cell division Arrests cell cycle at S and G1 phases by interfering with cyclins and CDK Activates and modulates ROS depending on apoptotic pathways through effects on p53, Bcl-2, caspases, IAP, and autophagy affecting MAPK and ERK Tumor development and metastasis Restrains neovascularization by downregulating expression of the endothelial growth factor Reinforces cell-tight junction Chemosensitization Potentiates cytotoxicity of different agents	[30,59,65,66,67,68,69,70,71,72]
	Anti-inflammatory	Reduces the release of proinflammatory cytokines TNF-α; IL-1β; and IL-6	[71,72]
Chelerythrine (III)	Adjuvant in COVID-19 treatment Anti-inflammatory	Prevents hyper-inflammatory immune response regulating signaling pathways mediated by Nrf2, NF-κB, and p38 MAPK	[73,74]
		Reduces protein kinase C-α/-β inhibitory activity, preventing cerebral vasospasm, eryptosis, and pulmonary inflammation and fibrosis	
	Antiviral	Viral RNA-intercalation	[73]
	Anticancer	Reduces phosphorylation of ERK and Akt, downplaying the activation of p53, B-cell Bcl-2, caspases, and PARP	[74]
Cheilanthifoline (IV)	Anti-inflammatory	Reduces the release of proinflammatory cytokines and anti-AChE	[75]
	Antimicrobial	Hinders expression of MRSA genes and disrupts membrane integrity	[76]

## Data Availability

Not applicable.
